# Draft genome sequence of *Klebsiella pneumoniae* KLEB-33: a convergent biofilm hyperforming multiresistant strain belonging to the emerging ST16 lineage harboring multiple hypervirulence genes

**DOI:** 10.1128/mra.01192-23

**Published:** 2024-03-01

**Authors:** Sergio Silva-Bea, Isidro García-Meniño, Sonia Rey, Manuel Romero, Javier Fernández, Jens A. Hammerl, Azucena Mora, Ana Otero

**Affiliations:** 1 Departamento de Microbioloxía e Parasitoloxía, Facultade de Bioloxía, Edificio CIBUS, Universidade de Santiago de Compostela, Santiago de Compostela, Spain; 2 Aquatic One Health Research Center (ARCUS), Universidade de Santiago de Compostela, Santiago de Compostela, Spain; 3 Departamento de Microbioloxía e Parasitoloxía, Laboratorio de Referencia de Escherichia coli (LREC), Universidade de Santiago de Compostela (USC), Lugo, Spain; 4 Instituto de Investigación Sanitaria de Santiago de Compostela (IDIS), Santiago, Spain; 5 Microbiology and Infectology Research Group, Galicia Sur Health Research Institute (IIS Galicia Sur), SERGAS-UVIGO, Vigo, Spain; 6 Microbiology Service, Complexo Hospitalario Universitario de Vigo (CHUVI), Sergas, Vigo, Spain; 7 Servicio de Microbiología, Hospital Universitario Central de Asturias (HUCA), Oviedo, Spain; 8 Grupo de Microbiología Traslacional, Instituto de Investigación Sanitaria del Principado de Asturias (ISPA), Oviedo, Spain; 9 Department Biological Safety, German Federal Institute for Risk Assessment (BfR), Berlin, Germany; Loyola University Chicago, Chicago, Illinois, USA

**Keywords:** *Klebsiella pneumoniae*, multidrug resistance, hypervirulence, convergent, OXA-48, ST16, biofilm formation, hypermucoviscous

## Abstract

The emergence of convergent *Klebsiella pneumoniae* strains showing multiresistance, characteristic of nosocomial pathotypes and hypervirulent traits typical of community-acquired isolates, makes them important models for studying *K. pneumoniae* pathogenesis. Here, we describe the convergent, multidrug-resistant KLEB-33 strain harboring several hypervirulence genes and make its genome available to the scientific community.

## ANNOUNCEMENT

The emergence of convergent strains of *Klebsiella pneumoniae* (1, 2), showing multidrug resistance typical of the classic pathotype (cKp) and hypervirulence traits (hvKp pathotype) (3, 4), prompted us to make public the draft genome of KLEB-33 (LREC_296), a multiresistant, hypermucoviscous, biofilm-producing convergent strain (5), harboring multiple hypervirulence genes, belonging to the sequence type ST16 (cgMLST 1705), an emerging high-risk lineage similar to other high-risk lineages spread worldwide, such as ST11 (6). The phenotypic and genotypic characteristics of this strain (Table 1) make it a strong candidate for use as a model for convergent multidrug-resistant strains of *K. pneumoniae*, and therefore, the availability of its genomic sequence will be a valuable resource for the scientific community.

**TABLE 1:** General genomic features of the *K. pneumoniae* KLEB-33 isolate

Acquired resistance determinants	Gene	Identity (%)	Reference coverage (nt)
Beta-lactam	*bla*_OXA-48_	100.00	798/798
	*bla*_SHV-1_	99.88	861/861
Fosfomycin	*fosA5*	95.00	420/420
Disinfectant	*oqxB*	98.89	3,153/3,153
	*oqxA*	99.15	1,176/1,176

Antimicrobial resistance-associated mutation	Mutation	Nucleotide change	Amino acid change
Carbapenem	mpK36 p.A217S	GCT-TCT	A-S
	mpK36 p.N218H	AAC-CAC	N-H
	mpK37 p.I70M	ATT-ATG	I-M
	mpK37 p.I128M	ATT-ATG	I-M
Cephalosporins	mpK36 p.N49S	AAC-AGC	N-S
	mpK36 p.L59V	CTT-GTA	L-V
	mpK36 p.L191S	CTG-AGC	L-S
	mpK36 p.F207W	TTC-TGG	F-W
	mpK36 p.D224E	GAT-GAG	D-E
	mpK36 p.L228V	CTG-GTT	L-V
	mpK36 p.E232R	GAA-CGT	E-R
	mpK36 p.T254S	ACT-TCT	T-S
Ciprofloxacin	gyrA p.S83F	TCC-TTC	S-F
	gyrA p.D87N	GAC-AAC	D-N
	parC p.E84K	GAA-AAA	E-K

Plasmid	Identity (%)	Reference coverage (nt)	Accession number and link
Col(MG828)	95.0	260/262	NC008486
Col(pHAD28)	92.37	131/131	KU674895
Col440II	100.0	270/282	CP023921
IncFIB(K)	98.75	560/560	JN233704
IncFII(K)	97.97	148/148	CP000648
IncL	99.85	661/661	JN626286
IncX4	98.88	712/712	FN543504

Plasmid sequence type
[K5:A-:B-]

Kaptive (capsule typing)	
K-locus	K51
O-locus	O3b

Mobile genetic element	Type	Length (nt)	E-value
ISEc9	Insertion sequence	1,652	0
IS26	Insertion sequence	820	0
ISKpn14	Insertion sequence	768	0
ISKpn14	Insertion sequence	768	0
ISEcl1	Insertion sequence	1,336	0
ISKpn33	Insertion sequence	2,526	0
ISKpn26	Insertion sequence	1,196	0
cn_50561_ISEcl1	Composite transposon	50,561	0

Hypervirulence determinants	Description	Identity-coverage
*peg-344*	Metabolite transport	100%-100%
*peg-589*	Metabolite transport	100%-100%
*peg-1631*	Metabolite transport	92%–100%
*iroN*	Aerobactin siderophores	68.94%-84.00%
*iutA*	Aerobactin siderophores	100%-100%
*pld1*	Endonuclease	100%-100%
*ompC*	Porine	99.18%-100%

Other virulence genes/clusters	
Siderophore biosynthesis/receptors	*entABCDEF* (enterobactin biosynthesis), *fepABCDG* (enterobactin receptor)
Fimbriae	*mrkABCDFHIJ* (fimbriae type 3), *fimABCDFGHI* (fimbriae type 1), *ecpA*
Capsule synthesis	*rcsACDF*
Other	*opmA* (outer membrane protein), *pal* (peptidoglycan-associated lipoprotein), *ybb*W (allantoin permease), *pld1*(phospholipase D family protein), *pgaABCD* (synthesis and translocation of poly B 1,6-N-acetyl-D-glucosamine adhesin)

The KLEB-33 isolate was recovered from a biliary drainage of a patient diagnosed with cholangitis at the Hospital Universitario Central de Asturias (Spain) in October 2018 as part of a study approved by the Institutional Ethics Committee (CEImPA 03/2018). The strain was recovered with a swab and isolated on Columbia agar 24 h/37°C in aerobiosis. Bacterial identification was performed by Matrix-Assisted Laser Desorption/Ionization - Time of Flight (MALDI-TOF) mass spectrometry. This OXA-48 carbapenemase-producing strain is resistant to ertapenem, ampicillin, amoxicillin-clavulanic acid, piperacillin-tazobactam, ciprofloxacin, and nitrofurantoin, as assessed by the broth microdilution susceptibility method with the MicroScan Neg MIC 57 panel (Walwaway Plus, Beckman Coulter) and confirmed by Etest (bioMérieux). European Committee on Antimicrobial Susceptibility Testing v13.0 clinical breakpoints and guidelines for *Enterobacteriaceae* were used for interpretation.

Genomic DNA (gDNA) was extracted from 1 mL Luria-Bertani (LB) culture (~10^9^ CFU/mL) incubated in aerobiosis, 180 rpm, 37°C/16–20 h, using the Purelink genomic DNA mini kit (Thermo Fisher Scientific, Waltham, USA). The quantity and quality of gDNA were assessed using Qubit (v4.0) and Nanodrop ND-1000 (Thermo Fisher Scientific), respectively. Whole Genome Sequencing (WGS) libraries were prepared using the Nextera DNA Flex library preparation kit, followed by paired-end sequencing (2 × 150 bp) on a NextSeq 500 device (Illumina, Inc., San Diego, CA, USA). Sequencing quality assessment and genome assembly of the raw reads were performed using the “Aquamis” pipeline (https://gitlab.com/bfr_bioinformatics/AQUAMIS/; settings: default; accession: October 2022) (7). The genome sequence comprises 5,466,901 nt assigned to 73 contigs (sequencing depth ≥ 80 per consensus base; N50: 273,927 nt; GC content: 57.24%; number of raw reads: 1,947,940). In the first version of the genome, the sequence is not closed (still segmented into contigs) and is not circular.

The “BakCharak” pipeline was run for initial *in silico* typing (https://gitlab.com/bfr_bioinformatics/bakcharak; settings: default; accession: October 2022), and the automated prokaryotic genome annotation pipeline (v6.3, National Center for Biotechnology Information) was used for genome annotation (8). Surface polysaccharide locus prediction (capsid and O-type/variant) and cgST determination were performed with Pathogenwatch (https://pathogen.watch). The presence of virulence and hypervirulence determinants in the genomic assemblies was predicted using the *Klebsiella* locus/sequence definitions database and the Bacterial Isolate Genome Sequence Database software of the Institut Pasteur (https://bigsdb.pasteur.fr/klebsiella/) (9). The presence of hypervirulence genes was confirmed using the BLASTn tool. General genomic characteristics and *in silico* analysis are summarized in Table 1.

## Data Availability

The Whole Genome Shotgun project of *K. pneumoniae* KLEB-33 (LREC_296) was deposited in GenBank under the accession no. JAPJTV000000000.1. The raw sequencing data are accessible in the SRA database with the following link (https://www.ncbi.nlm.nih.gov/sra/PRJNA891646), under the BioProject PRJNA891646. Sequencing raw reads and assembly data are linked to BioSample SAMN31340319. The SRA records can also be retrieved from the European Nucleotide Archive (ENA) (https://www.ebi.ac.uk/ena/browser/view/PRJNA891646). The version described in this paper is the first version.

## References

[1] Gonzalez-Ferrer S, Peñaloza HF, Budnick JA, Bain WG, Nordstrom HR, Lee JS, Van Tyne D. 2021. Finding order in the chaos: outstanding questions in Klebsiella pneumoniae pathogenesis. Infect Immun 89:e00693-20. doi:10.1128/IAI.00693-20 33558323 PMC8090965

[2] Lan P, Jiang Y, Zhou J, Yu Y. 2021. A global perspective on the convergence of hypervirulence and carbapenem resistance in Klebsiella pneumoniae. J Glob Antimicrob Resist 25:26–34. doi:10.1016/j.jgar.2021.02.020 33667703

[3] Russo TA, Marr CM. 2019. Hypervirulent Klebsiella pneumoniae. Clin Microbiol Rev 32:.1128 doi:10.1128/CMR.00001-19 PMC6589860 31092506

[4] Zhu J, Wang T, Chen L, Du H. 2021. Virulence factors in hypervirulent Klebsiella pneumoniae. Front Microbiol 12:642484. doi:10.3389/fmicb.2021.642484 33897652 PMC8060575

[5] Silva-Bea S, Romero M, Parga A, Fernández J, Mora A, Otero A. 2024. Comparative analysis of multidrug-resistant Klebsiella pneumoniae strains of food and human origin reveals overlapping populations. Int J Food Microbiol 413:110605. doi:10.1016/j.ijfoodmicro.2024.110605 38308879

[6] de Sales RO, Leaden L, Migliorini LB, Severino P. 2022. A comprehensive genomic analysis of the emergent Klebsiella pneumoniae ST16 lineage: virulence, antimicrobial resistance and a comparison with the clinically relevant ST11 strain. Pathogens 11:1394. doi:10.3390/pathogens11121394 36558729 PMC9781218

[7] Deneke C, Brendebach H, Uelze L, Borowiak M, Malorny B, Tausch SH. 2021. Species-specific quality control, assembly and contamination detection in microbial isolate sequences with AQUAMIS. Genes (Basel) 12:644. doi:10.3390/genes12050644 33926025 PMC8145556

[8] Tatusova T, DiCuccio M, Badretdin A, Chetvernin V, Nawrocki EP, Zaslavsky L, Lomsadze A, Pruitt KD, Borodovsky M, Ostell J. 2016. NCBI prokaryotic genome annotation pipeline. Nucleic Acids Res. 44:6614–6624. doi:10.1093/nar/gkw569 27342282 PMC5001611

[9] Jolley KA, Bray JE, Maiden MCJ. 2018. Open-access bacterial population genomics: BIGSdb software, the PubMLST.org website and their applications. Wellcome Open Res 3:124. doi:10.12688/wellcomeopenres.14826.1 30345391 PMC6192448

